# Behaviour-Related Scalar Habitat Use by Cape Buffalo (*Syncerus caffer caffer*)

**DOI:** 10.1371/journal.pone.0145145

**Published:** 2015-12-16

**Authors:** Emily Bennitt, Mpaphi Casper Bonyongo, Stephen Harris

**Affiliations:** 1 School of Biological Sciences, University of Bristol, Bristol, United Kingdom; 2 Okavango Research Institute, Maun, Botswana; University of Florida, UNITED STATES

## Abstract

Studies of habitat use by animals must consider behavioural resource requirements at different scales, which could influence the functional value of different sites. Using Cape buffalo (*Syncerus caffer caffer*) in the Okavango Delta, Botswana, we tested the hypotheses that behaviour affected use between and within habitats, hereafter referred to as macro- and microhabitats, respectively. We fitted GPS-enabled collars to fifteen buffalo and used the distances and turning angles between consecutive fixes to cluster the resulting data into resting, grazing, walking and relocating behaviours. Distance to water and six vegetation characteristic variables were recorded in sites used for each behaviour, except for relocating, which occurred too infrequently. We used multilevel binomial and multinomial logistic regressions to identify variables that characterised seasonally-preferred macrohabitats and microhabitats used for different behaviours. Our results showed that macrohabitat use was linked to behaviour, although this was least apparent during the rainy season, when resources were most abundant. Behaviour-related microhabitat use was less significant, but variation in forage characteristics could predict some behaviour within all macrohabitats. The variables predicting behaviour were not consistent, but resting and grazing sites were more readily identifiable than walking sites. These results highlight the significance of resting, as well as foraging, site availability in buffalo spatial processes. Our results emphasise the importance of considering several behaviours and scales in studies of habitat use to understand the links between environmental resources and animal behavioural and spatial ecology.

## Introduction

The way in which animals utilise the resources in their environments depends on the characteristics of the resources available to them at different scales [1]. Resource use at larger scales therefore restricts options at smaller scales and can affect the way in which animals interact with their environment [2]. Although researchers define macrohabitats according to broad structural characteristics, animals may distinguish between particular sites within those macrohabitats based on their functional value [3]. Characteristics such as woody components, herbaceous composition, soil type, and water availability vary between macrohabitats, but also within them [4]. The resulting microhabitats, defined here as sites with different structural characteristics within the same macrohabitat type, can be of disparate value to animals [5].

Macrohabitat characteristics can affect their suitability for use by animals for particular behaviours, highlighting the need to include behavioural requirements in analyses of resource use [6]. Some behaviours are highly time-consuming, e.g. foraging, but others can be crucial despite requiring only a small amount of time, such as drinking or salt licking [7]. Some macrohabitats may be more suited to particular behaviours than others because of their physical characteristics, e.g. resting in shady or sheltered locations [8].

Animal behaviour is identified most accurately through direct observation, but technological developments [2], combined with increasingly refined mathematical techniques [9], allow the use of data from GPS collars to distinguish between behaviours [10]. When foraging within a profitable site, animals typically move short distances with large turning angles [11]. Prolonged periods of very small movements indicate resting sites [12], whereas movements over larger distances with smaller turning angles occur when walking through unprofitable sites [13]. Sites used for particular behaviours can therefore be identified remotely, then visited to determine which environmental characteristics govern behaviour-specific site use [10].

Most habitat use studies focus on macrohabitats, and although microhabitat use has been analysed in small species [14, 15], relatively few studies have considered large herbivores [8, 16, 17, 18]. Also, most previous studies on microhabitat use have examined site use for specific behaviours, e.g. foraging [19], resting [8] or predator avoidance [20, 21], rather than differential behavioural use (but see [22, 23, 24]). Optimal sites for particular behaviours usually differ [25], so the use of specific microhabitats should be related to behaviour [22].

Large-bodied, gregarious bulk grazers are among the least selective herbivores [26], so there may be less ecological pressure for them to distinguish between microhabitats compared to smaller, more selective herbivores. However, identifying optimal sites for particular behaviours presumably would enhance the efficiency of their small-scale movements, as well as being beneficial at larger scales [27]. Relating macro- and microhabitat use by bulk grazers to their behaviour will improve understanding of their resource requirements at different scales, an important conservation issue given the key role of large herbivores in ecosystems as consumers and facilitators [28], but also as ecosystem engineers that can affect grass species diversity [29].

We used Cape buffalo (*Syncerus caffer caffer*) in the Okavango Delta, Botswana, to test the hypotheses that (i) macrohabitat use is related to behaviour, and (ii) variations in forage characteristics at the microhabitat scale elicit a behavioural response.

## Methods

### Study Area

**Fig 1:**
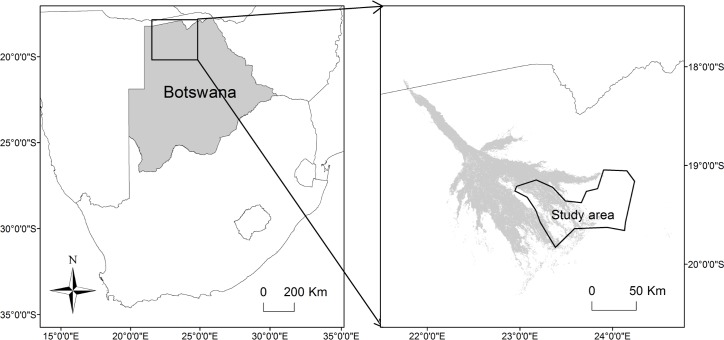
Location of the Okavango Delta and the study area in Botswana. Shading in the right-hand image shows the permanently flooded areas.

The Okavango Delta, located between E22.00-E24.00 and S18.50-S20.50 [30], is a flood-pulsed ecosystem with a dual moisture regime due to local annual rains and a delayed flooding response to rainfall further upstream in Angola [31]. We defined three seasons: the early flood season (April-July), when flood waters advanced; the late flood season (August-November), when flood waters receded; and the rainy season (December-March), when most rainfall occurred. The study site in the south-eastern Okavango Delta contained both flooded and dry regions (Fig 1), and covered approximately 5000 km^2^.

We defined ten macrohabitat types based on differences in woody and grass species composition observed during a 3-month pilot study, which were similar to previously described macrohabitats [32]. We developed a macrohabitat map from geo-referenced ortho-photographs (Okavango Research Institute) taken between 2001 and 2003. To test the accuracy of the map, we recorded 792 ground-truthing points in the seven macrohabitat types most intensively used by buffalo, i.e. a mean ± SD of 113.1 ± 32.5 (range 65–173) points per macrohabitat. The map represented the true macrohabitat type 88.1% of the time; accuracy was lowest for grassland (78.6%) and highest for riparian woodland (95.7%).

Secondary floodplain was wet for the whole year, so was mapped as a spatial layer identifying the location of permanent water. We used Google Earth (Google Inc., Mountain View, CA) to record the locations of every seasonal pan in the study area, and mapped them as a spatial layer identifying the locations of ephemeral water sources.

### Capture and Collaring

We fitted 15 female buffalo in different herds with Tellus Simplex 4D GPS-enabled satellite collars (Followit, Lindenberg, Sweden) programmed to record hourly GPS fixes, defined as geographical coordinates identified by latitude and longitude. The collars weighed 1.8 kg, 0.4% of the weight of the smallest cow we collared (450 kg). Weight was estimated from girth measurements using a growth curve developed for buffalo in Botswana (L Patterson 1978, unpublished report). We selected cows because they were more likely to retain their collars [33] and formed the core of mixed-sex breeding herds, so data from each cow were representative of the herd [34]. We carried out 24 darting operations (15 to fit collars, two to replace malfunctioning collars, seven to remove collars). We used a helicopter for 22 darting operations and a vehicle twice. Animals were immobilized with 8 mg A3080, reversed with Naltrexone (*N* = 13), or a combination of 10 mg M99, 40 mg Azaperone and 5000 i.u. Hyalase, reversed with 42 mg M5050 (*N* = 11).

### Ethics Statement

One of three experienced wildlife veterinarians registered with the government of Botswana carried out each darting operation under permit numbers EWT 3/3/8XXXVII 44 and EWT 8/36/4IV 62 from the Department of Wildlife and National Parks. All darted animals were adult females in good condition that were not obviously pregnant or with a young calf. Every effort was made to minimise the stress to darted buffalo and their herds. All buffalo recovered quickly from the darting operations; no ill effects were observed and they were all seen rejoining their herds.

Six collars dropped off and were recovered after the belting failed, seven animals were darted to remove collars at the end of the study, and two collars could not be recovered because they failed suddenly and ceased to emit the VHF signals used to locate the buffalo. All capture and handling procedures were approved by the University of Bristol Ethics Committee (UB/08/034) and conformed to the American Society of Mammalogists’ guidelines for the use of wild mammals in research [35]. All darting operations were carried out on government-owned protected land under control of the Department of Wildlife and National Parks, after permission had been obtained from concessionaires and all other relevant stake-holders. No protected or endangered species were involved in the research.

### Movement Data

Prior to deployment, we hung each collar at a height of 1 m for at least 100 hours and we took the mean position of these hourly test fixes as the reference position [36]. We measured the distance between each test fix and the reference position using the Point Distance tool in ArcGIS 10.0 (ESRI, Redlands, CA). The 95% circular error probability, the area containing 95% of fixes [37], was used to define the minimum distance threshold (MDT) for each collar, below which 95% of fixes could not be distinguished from stationary relocations [12]. The MDT varied with collar because of slight differences between them, in particular the improvements incorporated into later batches of collars. We calculated the distances and turning angles between temporally consecutive GPS fixes using the ‘Path, with distances and bearings’ extension (*http*:*//www*.*jennessent*.*com/downloads/Find_Path_online*.*pdf*) in ArcView 3.2 (ESRI, Redlands, CA).

We designated fixes ≤ MDT from the previous location as resting and fixes > MDT from the previous location as active [12], and then grouped active fixes into movement states based on their distances and turning angles using k-means cluster analysis [9]. We did not standardize the values for distance and turning angle because this would have given both measures equal weighting for the clustering procedure. Distance was more representative of behaviour because it defined the speed at which buffalo were moving: grazing buffalo must move at a speed conducive to feeding, though the path may be straight or tortuous, thereby yielding variable turning angles. The clustering algorithm produced three clusters consistent with movements at different spatial scales: grazing within a site, walking between sites, and relocating between ranges [34]. We defined sites as locations used by buffalo for particular behaviours, and identified by GPS coordinates. We visited those sites to sample the vegetation in a 50 m radius around the coordinates, which would have allowed for location error from the collars, while permitting us to stay close to the safety of the vehicle in a region with high densities of potentially dangerous animals.

We verified the accuracy of our behavioural identification method through direct observation of behaviours that were later related to GPS fixes, confirming that observed behaviours corresponded to remotely-recorded ones. Observations were conducted every time a herd containing a collared individual was located, approximately once per month, providing data across the seasons. We also observed signs related to each behaviour in the sampling sites: flattened areas with large amounts of faeces in resting sites; clean cuts on grass in grazing sites; and trampled game trails in walking sites. In addition, the proportion of time spent in each behaviour was similar to previous studies [38, 39]. Thus, each GPS fix identified a site where the buffalo were engaged in one of four behaviours i.e. resting, grazing, walking and relocating. However, relocating occurred infrequently and so we did not include it in the vegetation sampling analyses. We assigned a macrohabitat type to every fix after plotting it onto the macrohabitat map.

### Vegetation Sampling

From August 2008 to July 2010, we sampled 550 sites associated with resting, grazing and walking from macrohabitats in which buffalo spent > 10% of their time during the first year of data collection, hereafter referred to as seasonally-preferred macrohabitats. Increased water levels during the second year caused shifts in macrohabitat use [34] and created access problems for some sampling sites, particularly in secondary and tertiary floodplains. Insufficient samples were obtained to include these macrohabitats in the early flood vegetation sampling dataset, although they were included in the proportional behaviour analyses. We sampled no more than three sites per day of use per herd, with at least four hours between use and a minimum of 12 sites per behaviour within each macrohabitat and season (mean ± SD = 18.3 ± 4.2). Variation in vegetation characteristics within macrohabitats were used to distinguish between microhabitats, defined as locations differing in their vegetation characteristics, such as biomass and canopy cover, but not in their herbaceous and woody species composition. These sites were used by buffalo for a variety of behaviours and were from all over their home ranges, so were assumed to be representative of the macrohabitats. They were used to compare vegetation characteristics both between and within macrohabitats.

Within 50 m of the GPS coordinates recorded by the collars, we quantified grass biomass using a Disc Pasture Meter (DPM) [40], dropped 50 times at 1 m intervals along five randomly-placed 10 m transects. We avoided DPM drops on woody plants and forbs because the DPM was calibrated for herbaceous biomass only:

$$Y=-1633+1791\sqrt{X}$$ (1)

where *X* is the mean settling height of 50 DPM drops and *Y* is the biomass in kg/ha (WSW Trollope, CJ Hines, LA Trollope 2000, unpublished report). When sites were flooded, we calculated biomass from grass cut to just below the water surface, dried in the sun and oven-dried at 60°C for 24 hours. We added the dried weights from the four quadrats and multiplied them by 10 to convert biomass from g/m^2^ to kg/ha [34].

We recorded forage dispersion at each site using the distance from a measuring stick to the nearest grass tuft [41] at 1 m intervals along four randomly-placed 10 m transects. We measured species richness and calculated species composition by throwing a 0.5 × 0.5 m quadrat randomly four times, identifying each grass species and estimating percentage composition to the nearest 5%. We measured the distance between the ground and the flat portion of five leaves per species from different plants to give the mean leaf table height. We estimated canopy cover by taking four photographs with the camera pointing straight up at 10 m from the recorded GPS coordinate in each of the cardinal directions. The percentage canopy cover was calculated using a grid superimposed on the image, and a mean value was generated from the four photographs. We used the water-related raster layers to calculate distance to water for each fix; distance to permanent water was used during the early and late flood seasons, and distance to the closest ephemeral or permanent water source was used during the rainy season.

From August 2008 to July 2009, we cut samples from all the grass species at each visited site to determine the species-specific mean proportion of leaf in different seasons. Up to five tufts in the 0.5 × 0.5 m quadrats were cut to within 1 cm of the ground, dried in the sun, oven-dried at 60°C for 24 hours and separated into leaf and stem, each of which was weighed to the nearest 0.01 g. Leaf to stem ratio is frequently used to indicate plant quality because it is linked to maturity stage and crude protein content [42], but some samples had very small leaf or stem components and so we used the mean percentage leaf composition of each species of grass. This was scored for every habitat type and season as high (> 66.6%), intermediate (33.3–66.6%) or low (< 33.3%). These species-specific scores were combined with the species composition data to produce a palatability index for each site.

We calculated one value per site for each of the following independent variables: biomass, tuft dispersion (dispersion), species richness (richness), mean leaf table height (height), palatability index (palatability), canopy cover (canopy) and distance to water (water). These vegetation characteristics described the abundance (biomass, dispersion and height) and quality (palatability and richness) of the herbaceous layer, and the physical attributes of the site (canopy and water). One or more variables within each of these three groups of characteristics were expected to differ between macrohabitats and between microhabitats [4, 43, 44].

### Statistical Analyses

Using the criteria defined by the clustering algorithm and the macrohabitat map, we allocated a behaviour and macrohabitat type to each fix. We then calculated the proportion of time spent engaging in each behaviour by buffalo in each season, and in every seasonally-preferred macrohabitat. We used the “compositions” package in R v3.1.0 [45] to transform the data into log-ratios, then analysed them using Multivariate Analyses of Variance (MANOVAs). This enabled us to determine whether buffalo changed their proportional behaviour in different seasons and macrohabitat types, with the latter indicating behaviour-related macrohabitat use. Univariate Analyses of Variance (ANOVAs) identified the proportional behaviours differing between seasons and macrohabitat types.

To standardize the seven vegetation characteristic variables, we subtracted the sample mean from each value and divided by the standard deviation of the sample. This improved homogeneity of variance and accounted for the use of different units of measurement without altering the distributions of the independent variables [46].

We used MLwiN v. 2.33 [47] to develop mixed binomial and multinomial logistic regression models with logit link functions to identify the factors that differed between macrohabitats and between microhabitats. We expected patterns of macro- and microhabitat use to vary between individual buffalo, which sometimes occupied spatially-distinct areas, so we included the random effect of individual buffalo in the macro- and microhabitat analyses. We also wanted to account for the possible effect of unbalanced sampling of microhabitats used for different behaviours on the vegetation characteristics sampled in the macrohabitats, so we included the random effect of microhabitat in the macrohabitat analyses.

A mixed binomial logistic regression model was only used for macrohabitats in the early flood season (Eq 2), when only two seasonally-preferred macrohabitat types were accessible. Grassland was selected as the reference macrohabitat type because it was the most common macrohabitat type in the study area.

$$\mathrm{logit}(\pi_{ij})=\beta_{1}x1_{ij}+\ldots +\beta_{n}xn_{ij}$$ (2)

where *y*_*ij*_ is the categorical response for individual *i* in microhabitat *j*, and we denote the probability of being in grassland by π_ij_; *x1 –xn* are vegetation characteristics.

Mixed multinomial logistic regression models were used to identify vegetation characteristics differing between the other nine seasonal macrohabitats in the late flood and rainy seasons (Eq 3). Models with different contrasts were used to compare macrohabitats. Reference macrohabitat categories were identified based on their prevalence across the study area, with the exception of mixed shrubland. Although mixed shrubland covered a large proportion of the study area, it was not present in every collared buffalo home range [34], so it was used as a comparison macrohabitat.

$$\log(\frac{\pi_{ij}^{\left(s\right)}}{\pi_{ij}^{\left(t\right)}})=\beta_{0}^{\left(s\right)}+\beta_{1}^{\left(s\right)}x1_{ij}+\ldots +\beta_{n}^{\left(s\right)}xn_{ij}+u_{ij}^{\left(s\right)}$$ (3)

where *y*_*ij*_ is the categorical response for individual *i* in microhabitat *j*, and we denote the probability of being in macrohabitat *s* by $\pi_{ij}^{\left(s\right)}$; *t* is the total number of categories; *x1 –xn* are vegetation characteristics; and $u_{ij}^{\left(s\right)}$ is a contrast-specific random effect.

Mixed multinomial logistic regression models were used to identify vegetation characteristics differing between each of three microhabitats used for different behaviours in the ten seasonally-preferred macrohabitats (Eq 4). Models with different contrasts were used to compare microhabitats. Microhabitats where buffalo rested were used as the main reference category because buffalo spent several consecutive hours there; microhabitats that buffalo walked through and spent very little time in were selected as comparison microhabitats.

$$\log(\frac{\pi_{i}^{\left(s\right)}}{\pi_{i}^{\left(t\right)}})=\beta_{0}^{\left(s\right)}+\beta_{1}^{\left(s\right)}x1_{i}+\ldots +\beta_{n}^{\left(s\right)}xn_{i}+u_{i}^{\left(s\right)}$$ (4)

where *y*_*i*_ is the categorical response for individual *i*, and we denote the probability of being in microhabitat *s* by $\pi_{i}^{\left(s\right)}$; *t* is the total number of categories; *x1 –xn* are vegetation characteristics; and $u_{i}^{\left(s\right)}$ is a contrast-specific random effect.

We fitted fully saturated models containing all seven vegetation characteristics to differentiate between macrohabitats and between microhabitats. However, no sampling sites in secondary floodplain during the late flood season had any canopy cover, and so, to avoid complete separation, this was left out of the late flood season macrohabitat model and the secondary floodplain microhabitat model [46]. We developed *a priori* model sets based on combinations of the vegetation characteristics identifying abundance (biomass, height and dispersion) and quality (palatability and richness) of the herbaceous layer, and physical attributes of the sampling site (canopy and water). We ran models with all possible combinations of variables within those broader groups of characteristics, and used Akaike’s Information Criterion (AIC) [48] to identify the model with the best fit. The same set of models was used for macrohabitat and microhabitat datasets. The models were used to predict the percentage of sites assigned to each macrohabitat and microhabitat type, then compared to the actual percentage as a measure of model accuracy. Means are presented ± SD.

## Results

The macrohabitat types most prevalent in the study area were grassland (22.0%), mixed shrubland (15.9%), dense mopane woodland (15.6%), riparian woodland (13.9%), secondary floodplain (13.2%), open mopane woodland (10.2%) and tertiary floodplain (6.3%). Vegetation characteristics varied between macrohabitats (Table 1).

**Table 1:** Vegetation characteristics in different seasonally-preferred macrohabitats.

Season	Macrohabitat	Biomass	Richness	Palatability	Dispersion	Height	Distance to water	Canopy cover
Early flood	Grassland	2650 ± 1012	2.7 ± 1.2	0.57 ± 0.16	7.8 ± 8.1	17.0 ± 7.2	1959 ± 2426	± 0.04
	Riparian woodland	2166 ± 1098	2.5 ± 1.2	0.64 ± 0.23	15.0 ± 14.0	17.2 ± 9.2	1205 ± 1827	0.21 ± 0.24
Late flood	Grassland	2386 ± 1032	3.2 ± 1.2	0.66 ± 0.14	7.7 ± 6.6	14.4 ± 7.2	1208 ± 1716	± 0.04
	Riparian woodland	1114 ± 806	1.9 ± 1.3	0.41 ± 0.28	30.3 ± 27.7	11.0 ± 8.9	551 ± 1103	0.22 ± 0.21
	Secondary floodplain	3189 ± 2152	2.4 ± 1.1	0.48 ± 0.20	7.2 ± 8.8	16.0 ± 10.5	114 ± 393	0.00 ± 0.00
	Tertiary floodplain	2824 ± 1034	2.1 ± 1.0	0.61 ± 0.07	3.2 ± 3.9	14.3 ± 6.8	275 ± 665	0.01 ± 0.05
Rainy	Grassland	2348 ± 666	3.5 ± 1.3	0.69 ± 0.19	7.5 ± 5.4	16.1 ± 5.4	315 ± 235	0.03 ± 0.08
	Dense mopane woodland	1166 ± 795	2.9 ± 1.3	0.72 ± 0.20	21.6 ± 15.6	15.8 ± 7.7	332 ± 783	0.15 ± 0.15
	Open mopane woodland	894 ± 710	2.3 ± 1.2	0.73 ± 0.19	23.7 ± 18.5	14.9 ± 7.1	225 ± 215	0.44 ± 0.18
	Mixed shrubland	2122 ± 693	2.6 ± 1.3	0.53 ± 0.23	13.9 ± 7.0	20.1 ± 8.1	791 ± 1017	0.06 ± 0.10

### Movement Data

Buffalo were collared for three to 16 months, after which collars failed or were removed. The clustering technique consistently identified similar distances and turning angles for the behaviour categories, although variation in MDT affected the cut-off point between resting and active behaviours (Table 2). Individual variation in movement patterns affected the mean values for distance and turning angle, highlighting the need to consider individuals separately. The location error of the GPS fixes was 17.2 ± 18.7 m, *N* = 3524, mean collar range 10.5–34.1 m. The percentage success for fix attempts by the collars varied between animals (82.0 ± 4.6%, *N* = 15, range 62.1–91.2%) and seasons. Although it was similar in the early (87.2 ± 5.8%, *N* = 14) and late flood seasons (87.8 ± 6.9%, *N* = 15), it was lower in the rainy season (75.6 ± 13.5%, *N* = 15), when buffalo spent more time in wooded habitats [34], where extensive canopy cover could have affected communication between the collars and satellites [36].

**Table 2:** Mean distances and turning angles defining behaviour, calculated by k-means cluster analysis on consecutive GPS fixes.

Buffalo	Number of consecutive fixes	Mean distance (m) ± SD				Mean turning angle (°) ± SD			
		Rest	Graze	Walk	Relocate	Rest	Graze	Walk	Relocate
B1	758	24 ± 18	222 ± 107	668 ± 171	1788 ± 620	85 ± 54	67 ± 48	57 ± 46	36 ± 36
B2	2010	22 ± 14	176 ± 83	528 ± 136	1244 ± 373	96 ± 54	73 ± 51	63 ± 47	54 ± 51
B3	6604	22 ± 14	190 ± 93	559 ± 141	1266 ± 365	96 ± 53	61 ± 48	53 ± 45	43 ± 42
B4	6614	22 ± 14	200 ± 101	629 ± 177	1709 ± 540	97 ± 53	67 ± 50	54 ± 46	46 ± 48
B5	8670	31 ± 22	258 ± 109	689 ± 177	1700 ± 576	96 ± 53	67 ± 50	54 ± 45	42 ± 41
B6	5768	25 ± 18	233 ± 113	709 ± 206	1966 ± 528	95 ± 55	61 ± 48	50 ± 44	40 ± 32
B7	5876	20 ± 13	208 ± 106	636 ± 169	1560 ± 646	99 ± 54	71 ± 51	57 ± 45	43 ± 38
B8	6365	31 ± 25	272 ± 108	679 ± 155	1453 ± 443	94 ± 55	58 ± 46	51 ± 42	47 ± 43
B9	7322	22 ± 16	247 ± 125	760 ± 203	1933 ± 644	97 ± 53	65 ± 50	53 ± 45	42 ± 41
B10	2080	19 ± 13	194 ± 107	666 ± 216	2263 ± 588	104 ± 53	76 ± 53	59 ± 49	21 ± 28
B11	5711	16 ± 9	204 ± 122	692 ± 196	1702 ± 516	99 ± 53	63 ± 50	48 ± 42	35 ± 37
B12	5809	18 ± 13	210 ± 98	579 ± 134	1215 ± 396	95 ± 54	59 ± 47	50 ± 42	42 ± 39
B13	6178	14 ± 8	173 ± 108	623 ± 172	1535 ± 490	99 ± 53	68 ± 51	51 ± 43	48 ± 42
B14	1973	24 ± 17	256 ± 123	725 ± 188	1761 ± 566	94 ± 53	58 ± 47	47 ± 41	43 ± 39
B15	3818	33 ± 26	269 ± 114	741 ± 194	1912 ± 660	97 ± 54	63 ± 48	57 ± 48	41 ± 43

### Proportional Behaviour

**Fig 2:**
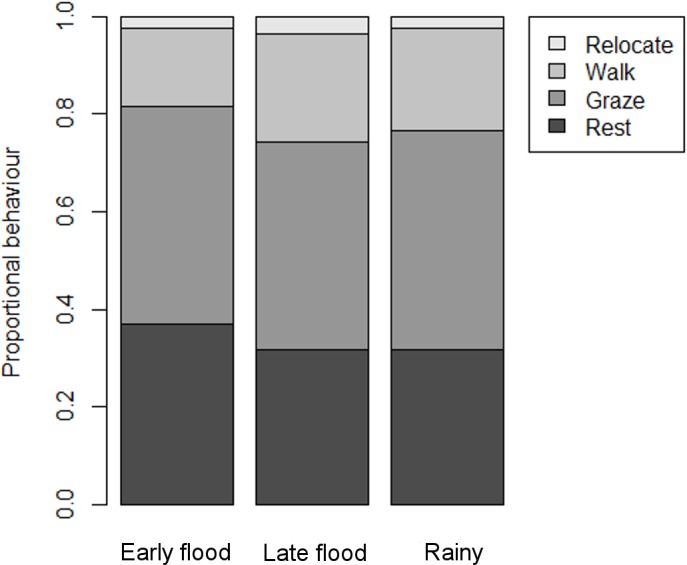
Seasonal variation in behaviour of 14, 15 and 15 female buffalo, respectively.

Seasonal behaviour data were generated from 14, 15 and 15 buffalo in the early flood, late flood and rainy seasons, respectively. MANOVAs showed that there was a significant difference between proportional behaviour during the early and late flood seasons (Pillai_1,24_ = 0.499, p = 0.002), but not between the early flood and rainy seasons (Pillai_1,24_ = 0.279, p = 0.086), nor between the late flood and rainy seasons (Pillai_1,25_ = 0.209, p = 0.194) (Fig 2). Buffalo walked more during the late flood (F_1,27_ = 8.443, p = 0.007) than the early flood season, but there was no difference in the time spent resting (F_1,27_ = 3.332, p = 0.079), grazing (F_1,27_ = 0.323, p = 0.574) or relocating (F_1,27_ = 1.703, p = 0.203). Grazing formed the largest part of the time budget, accounting for 43.9 ± 5.1% of proportional behaviour.

To avoid possible biases from a small number of fixes, data on proportional behaviour in macrohabitats were only used if a collar had recorded at least 20 fixes within a particular macrohabitat. For the same reason, we only analysed proportional behaviour within seasonally-preferred macrohabitats. Proportional behaviour varied more during the early and late flood seasons than during the rainy season (Table 3).

**Table 3:** MANOVA results from comparisons of proportional behaviour in seasonally-preferred macrohabitats.

Season	Macrohabitat A	Macrohabitat B	MANOVA result
Early flood	Grassland	Riparian woodland	Pillai_1,22_ = 0.393, **p = 0.022**
		Secondary floodplain	Pillai_1,17_ = 0.521, **p = 0.010**
		Tertiary floodplain	Pillai_1,21_ = 0.133, p = 0.535
	Riparian woodland	Secondary floodplain	Pillai_1,18_ = 0.731, **p<0.001**
		Tertiary floodplain	Pillai_1,22_ = 0.388, **p = 0.024**
	Secondary floodplain	Tertiary floodplain	Pillai_1,17_ = 0.543, **p = 0.007**
Late flood	Grassland	Riparian woodland	Pillai_1,25_ = 0.452, **p = 0.004**
		Secondary floodplain	Pillai_1,24_ = 0.484, **p = 0.002**
		Tertiary floodplain	Pillai_1,25_ = 0.059, p = 0.815
	Riparian woodland	Secondary floodplain	Pillai_1,24_ = 0.724, **p<0.001**
		Tertiary floodplain	Pillai_1,25_ = 0.502, **p = 0.001**
	Secondary floodplain	Tertiary floodplain	Pillai_1,24_ = 0.387, **p = 0.016**
Rainy	Grassland	Dense mopane woodland	Pillai_1,25_ = 0.236, p = 0.137
		Open mopane woodland	Pillai_1,25_ = 0.250, p = 0.114
		Mixed shrubland	Pillai_1,23_ = 0.060, p = 0.828
	Dense mopane woodland	Open mopane woodland	Pillai_1,25_ = 0.193, p = 0.235
		Mixed shrubland	Pillai_1,23_ = 0.336, **p = 0.044**
	Open mopane woodland	Mixed shrubland	Pillai_1,23_ = 0.240, p = 0.160

Significant results are in bold.

**Fig 3:**
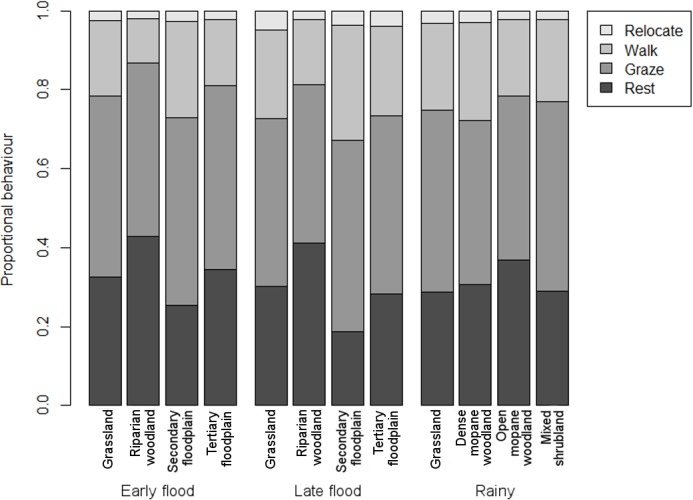
Seasonal variation in behaviour between seasonally-preferred macrohabitats.

The only macrohabitats in which buffalo proportional behaviour was not significantly different during the two flood seasons were grassland and tertiary floodplain, both of which were open and provided similar resources and environmental conditions. Most of the variation in proportional behaviour between macrohabitats was caused by trade-offs between time spent resting and walking (Table 4). Time spent grazing showed no variation between macrohabitats, in keeping with results from seasonal comparisons. Proportional behaviour in different macrohabitats was similar during the flood seasons (Fig 3), indicating that the functional value of macrohabitats for particular behaviours was not driven by season alone.

**Table 4:** ANOVA results identifying behaviours causing differences in proportional behaviour between seasonally-preferred macrohabitats.

Season	MacrohabitatA	MacrohabitatB	Behaviour			
			Rest	Graze	Walk	Relocate
Early flood	Grassland	Riparian woodland	F_1,25_ = 9.404, **p = 0.005**	F_1,25_ = 0.013, p = 0.517	F_1,25_ = 14.568, **p<0.001**	F_1,25_ = 0.105, p = 0.749
		Secondary floodplain	F_1,20_ = 5.303, **p = 0.032**	F_1,20_ = 0.023, p = 0.882	F_1,20_ = 0.049, p = 0.827	F_1,20_ = 3.835, p = 0.064
	Riparian woodland	Secondary floodplain	F_1,21_ = 16.254, **p<0.001**	F_1,21_ = 0.338, p = 0.567	F_1,21_ = 4.820, **p = 0.040**	F_1,21_ = 1.406, p = 0.249
		Tertiary floodplain	F_1,25_ = 5.404, **p = 0.029**	F_1,25_ = 0.482, p = 0.494	F_1,25_ = 5.876, **p = 0.023**	F_1,25_ = 1.359, p = 0.255
	Secondary floodplain	Tertiary floodplain	F_1,20_ = 6.098, **p = 0.023**	F_1,20_ = 0.002, p = 0.964	F_1,20_ = 0.567, p = 0.460	F_1,20_ = 0.012, p = 0.913
Late flood	Grassland	Riparian woodland	F_1,28_ = 15.436, **p<0.001**	F_1,28_ = 0.747, p = 0.395	F_1,28_ = 7.876, **p = 0.009**	F_1,28_ = 4.229, **p = 0.049**
		Secondary floodplain	F_1,27_ = 21.835, **p<0.001**	F_1,27_ = 3.806, p = 0.062	F_1,27_ = 5.247, **p = 0.030**	F_1,27_ = 0.424, p = 0.521
	Riparian woodland	Secondary floodplain	F_1,27_ = 58.352, **p<0.001**	F_1,27_ = 8.146, **p = 0.008**	F_1,27_ = 28.617, **p<0.001**	F_1,27_ = 8.165, **p = 0.008**
		Tertiary floodplain	F_1,28_ = 18.713, **p<0.001**	F_1,28_ = 4.182, p = 0.050	F_1,28_ = 11.523, **p = 0.002**	F_1,28_ = 7.788, **p = 0.009**
	Secondary floodplain	Tertiary floodplain	F_1,27_ = 13.786, **p<0.001**	F_1,27_ = 1.163, p = 0.290	F_1,27_ = 6.165, **p = 0.020**	F_1,27_ = 0.315, p = 0.579
Rainy	Dense mopane woodland	Mixed shrubland	F_1,26_<0.001, p = 0.985	F_1,26_ = 9.967, **p = 0.004**	F_1,26_ = 2.735, p = 0.110	F_1,26_ = 0.582, p = 0.453

Significant results are in bold.

### Multilevel Binomial and Multinomial Logistic Regressions

In the majority of cases, fully saturated models containing all seven vegetation characteristics were the most parsimonious. However, the best fitting early flood macrohabitat model, early flood riparian woodland microhabitat model, late flood grassland microhabitat model, and late flood riparian woodland model did not include height or richness, richness, water, and palatability, respectively. Despite being included in the most parsimonious models, not all of the vegetation characteristics had a significant effect.

**Fig 4:**
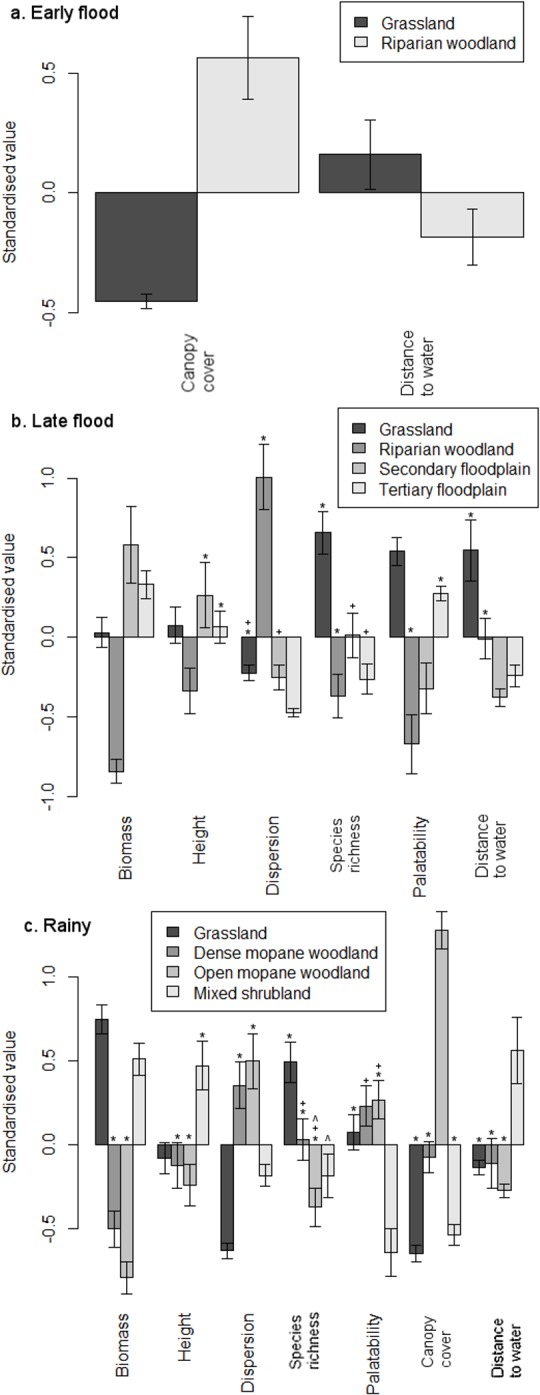
Variables discriminating between seasonally-preferred macrohabitats during the (a) early flood, (b) late flood and (c) rainy seasons. Values marked with (*), (+), or (^) were not significantly different from each other.

The models could distinguish between macrohabitats in every season, based on two, six and six variables in the early flood, late flood and wet seasons, respectively (Table 5; Fig 4). All of the variables differed significantly between at least two macrohabitats, and the high significance of most of the parameters indicated that the macrohabitats we defined were clearly distinct in terms of their vegetation characteristics. Residual plots from the models showed normal distributions.

**Table 5:** Vegetation characteristics differing significantly between seasonally-preferred macrohabitats.

Season	Reference habitat	Comparison habitat	Parameter	Estimate	Χ^2^	P-value
Early flood	Grassland	Riparian woodland	Canopy	2.393	16.004	<0.001
			Distance to water	-0.844	4.222	0.041
Late flood	Grassland	Riparian woodland	Biomass	-1.178	18.727	<0.001
			Height	0.414	4.426	0.022
			Palatability	-1.478	21.390	<0.001
		Secondary floodplain	Biomass	1.400	26.152	<0.001
			Height	-0.515	5.493	0.019
			Richness	-1.479	53.393	<0.001
			Palatability	-1.860	77.632	<0.001
			Distance to water	-1.071	52.599	<0.001
		Tertiary floodplain	Biomass	1.238	19.007	<0.001
			Height	-0.525	8.090	0.004
			Dispersion	-1.874	53.676	<0.001
			Richness	-1.657	82.023	<0.001
			Palatability	-1.399	39.184	<0.001
			Distance to water	-0.871	24.893	<0.001
	Riparian woodland	Secondary floodplain	Biomass	1.999	50.754	<0.001
			Height	-0.568	6.447	0.011
			Dispersion	-1.545	15.974	<0.001
			Richness	-1.013	22.559	<0.001
			Palatability	-0.890	16.479	<0.001
			Distance to water	-0.855	31.396	<0.001
		Tertiary floodplain	Biomass	2.696	103.898	<0.001
			Height	-0.703	14.577	<0.001
			Dispersion	-1.292	29.180	<0.001
			Richness	-1.207	43.216	<0.001
			Distance to water	-0.650	13.376	<0.001
	Secondary floodplain	Tertiary floodplain	Biomass	-0.747	10.426	0.001
			Dispersion	-2.691	48.619	<0.001
			Palatability	1.142	31.109	<0.001
			Distance to water	0.453	11.907	0.001
Rainy	Grassland	Dense mopane woodland	Biomass	-1.539	51.388	<0.001
			Height	1.000	20.730	<0.001
			Dispersion	2.198	54.794	<0.001
			Palatability	1.030	34.360	<0.001
		Open mopane woodland	Biomass	-0.984	27.796	<0.001
			Height	0.533	9.900	0.002
			Dispersion	1.578	30.634	<0.001
			Canopy	2.901	116.600	<0.001
		Mixed shrubland	Biomass	-0.673	9.435	0.002
			Height	1.230	35.703	<0.001
			Dispersion	0.639	4.821	0.028
			Richness	-0.527	13.007	<0.001
			Palatability	-0.519	8.344	0.004
			Distance to water	0.855	4.932	0.026
	Dense mopane woodland	Open mopane woodland	Canopy	1.785	58.682	<0.001
			Distance to water	0.827	5.594	0.018
		Mixed shrubland	Biomass	1.300	29.891	<0.001
			Dispersion	-0.681	13.542	<0.001
			Richness	-0.576	12.538	<0.001
			Palatability	-1.040	28.998	<0.001
			Distance to water	2.897	38.546	<0.001
	Open mopane woodland	Mixed shrubland	Biomass	1.252	28.064	<0.001
			Dispersion	-0.495	7.238	0.007
			Palatability	-0.871	20.447	<0.001
			Canopy	-1.899	81.801	<0.001
			Distance to water	1.518	10.124	0.001

Physical attributes of sampling sites were best able to distinguish between macrohabitats in the early flood season, indicating that herbaceous characteristics were relatively uniform during that period. During the late flood season, open macrohabitats (grassland, secondary and tertiary floodplain) were superficially similar in appearance, but differed in measures of their herbaceous abundance and quality, as well as physical attributes. Riparian woodland was characterised by low levels of forage abundance, whereas measures of vegetation quality were highest in grassland. During the rainy season, vegetation characteristics in mixed shrubland were most different from those in the other macrohabitats. Vegetation abundance was lower in both mopane woodland macrohabitats than in grassland and mixed shrubland, and canopy cover was highest in open mopane woodland.

Group membership as predicted by the macrohabitat models was similar to the actual proportion of samples taken from each macrohabitat, indicating that the predictive power of the models was substantial (Table 6). The models were well able to distinguish between macrohabitats based on the recorded vegetation characteristics.

**Table 6:** Percentages of samples assigned to seasonally-preferred macrohabitats from actual data and from multilevel model predictions.

Season	Macrohabitat	Predicted percentage	Actual percentage
Early flood	Grassland	54.4	54.5
	Riparian woodland	45.6	45.5
Late flood	Grassland	24.8	26.9
	Riparian woodland	25.4	24.9
	Secondary floodplain	24.5	16.9
	Tertiary floodplain	25.3	25.3
Rainy	Grassland	25.4	27.3
	Dense mopane woodland	25.7	23.4
	Open mopane woodland	24.6	25.5
	Mixed shrubland	24.2	23.8

The models could distinguish between microhabitats used for some of the different behaviours in every seasonal macrohabitat type, but not between microhabitats used for each of the different behaviours (Table 7). Models with the lowest AIC values included at least six predictors, but not all of those had a significant effect on the dependent variable, and levels of significance were lower than for parameters distinguishing between macrohabitats. Pair-wise differences were significant nine, four and seven times between resting and grazing, resting and walking, and grazing and walking sites, respectively. Height and dispersion were the characteristics that were most and least frequently able to distinguish between sites, respectively. No measures of herbaceous abundance or quality, or of physical attributes, were consistently able to differentiate between pairs of microhabitats used for different behaviours. Residual plots from the models showed normal distributions.

**Table 7:** Vegetation characteristics differing significantly between microhabitats used for different behaviours.

Season	Habitat	Reference behaviour	Comparison behaviour	Parameter	Estimate	Χ^2^	P-value
Early flood	Grassland	Rest	Graze	Biomass	0.842	4.153	0.042
				Dispersion	0.822	6.194	0.013
		Graze	Walk	Dispersion	-0.733	3.943	0.047
				Palatability	-1.467	7.508	0.006
				Canopy	-3.238	5.529	0.019
				Distance to water	2.222	14.111	<0.001
	Riparian woodland	Rest	Graze	Height	0.810	3.884	0.049
		Graze	Walk	Height	-0.943	4.525	0.033
Late flood	Grassland	Rest	Graze	Dispersion	1.110	5.731	0.017
	Riparian woodland	Rest	Graze	Height	-1.022	6.540	0.011
				Richness	1.873	11.537	0.001
				Canopy	1.168	9.831	0.002
		Graze	Walk	Canopy	-0.726	4.170	0.041
	Secondary floodplain	Rest	Graze	Biomass	-2.921	7.099	0.008
				Height	2.330	9.814	0.002
				Palatability	2.015	4.285	0.038
				Distance to water	0.929	5.766	0.016
		Rest	Walk	Height	2.155	9.764	0.002
				Distance to water	1.033	7.284	0.007
	Tertiary floodplain	Rest	Graze	Height	-1.406	11.582	0.001
				Richness	1.173	13.311	<0.001
				Palatability	0.823	4.645	0.031
		Rest	Walk	Height	-1.557	13.506	<0.001
				Richness	1.222	14.741	<0.001
				Palatability	1.001	6.619	0.010
		Graze	Walk	Biomass	-1.516	8.532	0.003
Rainy	Grassland	Rest	Graze	Richness	0.679	5.423	0.020
		Rest	Walk	Richness	0.723	6.166	0.013
	Dense mopane woodland	Rest	Graze	Distance to water	0.608	3.937	0.047
		Rest	Walk	Richness	0.627	4.056	0.044
		Graze	Walk	Biomass	-1.241	11.785	0.001
				Palatability	0.854	6.374	0.012
				Canopy	-1.356	18.763	<0.001
				Distance to water	-0.712	4.970	0.026
	Open mopane woodland	Rest	Graze	Height	0.987	8.824	0.003
				Palatability	-0.816	6.925	0.008
		Rest	Walk	Biomass	0.732	4.236	0.040
		Graze	Walk	Height	-1.729	16.688	<0.001
				Canopy	-1.037	10.179	0.001
				Distance to water	-1.616	10.186	0.001
	Mixed shrubland	Graze	Walk	Richness	-0.939	7.207	0.007
				Canopy	-0.942	5.655	0.017

**Fig 5:**
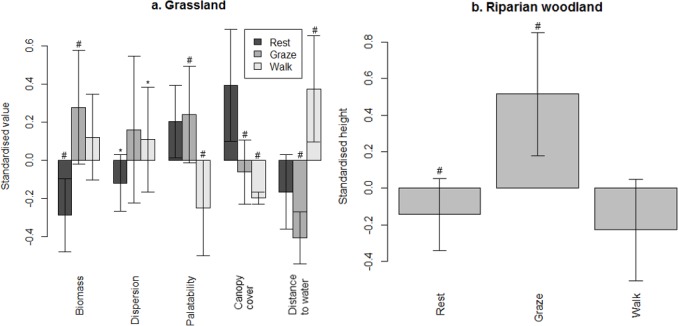
Variables discriminating between microhabitats used for different behaviours during the early flood season. Values marked with (#) and (*) were significantly and not significantly different from each other, respectively.

In both macrohabitats during the early flood season, measures of herbaceous abundance were lowest in resting sites and highest in grazing sites (Fig 5). Grazing sites also had higher quality forage than sites that were walked through, which indicated avoidance.

**Fig 6:**
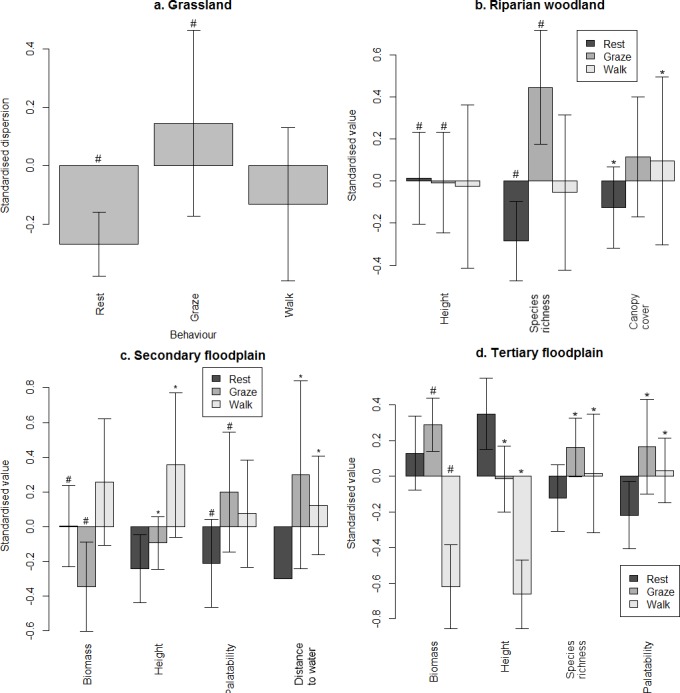
Variables discriminating between microhabitats used for different behaviours during the late flood season. Values marked with (#) and (*) were significantly and not significantly different from each other, respectively.

During the late flood season, resting sites in grassland had more abundant vegetation than grazing sites (Fig 6), possibly because buffalo would have been better able to select particular forage in the latter. In all other macrohabitats, grazing sites had high measures of forage quality. In secondary floodplain, walking sites had the highest measures of herbaceous abundance, but these may have included unpalatable sedges that buffalo would have avoided grazing [49]. In contrast, walking sites in tertiary floodplain were characterised by the lowest measures of abundance.

**Fig 7:**
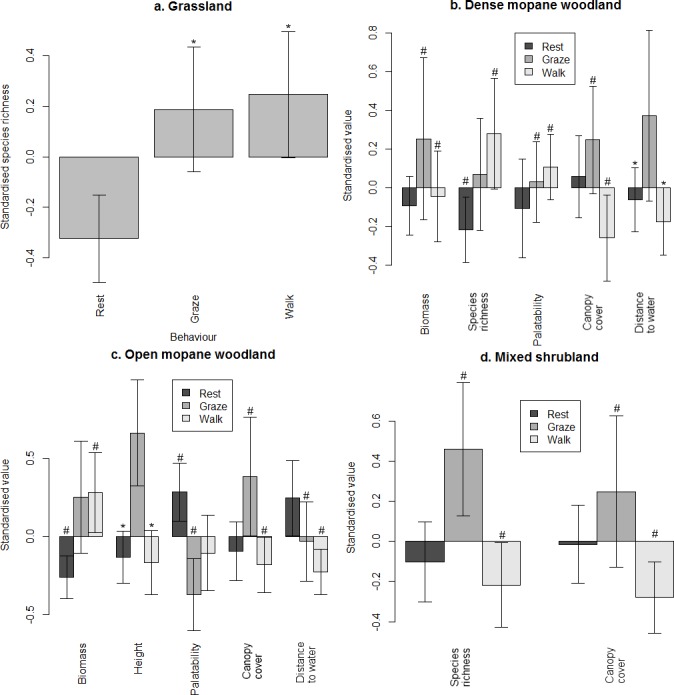
Variables discriminating between microhabitats used for different behaviours during the rainy season. Values marked with (#) and (*) were significantly and not significantly different from each other, respectively.

During the rainy season, canopy cover did not differ between resting sites and the microhabitats used for grazing or walking, so resting site use was based on other characteristics (Fig 7). Resting sites consistently had the lowest measures of forage abundance, and grazing sites were characterised by either high abundance or high quality.

Models distinguishing between microhabitats used for different behaviours showed a much lower level of accuracy in predicting group membership than the macrohabitat models, although the distribution of microhabitat samples was more skewed than the macrohabitat samples (Table 8). This indicated that the differences between microhabitats used for different behaviours were less pronounced than the differences between macrohabitats, in keeping with the lower levels of variation in vegetation characteristics between microhabitats than between macrohabitats.

**Table 8:** Percentages of samples assigned to behaviours from actual data and from multilevel model predictions.

Season	Macrohabitat	Percentage	Rest	Graze	Walk
Early flood	Grassland	Predicted	38.6	37.8	23.5
		Actual	44.4	22.2	33.3
	Riparian woodland	Predicted	34.6	32.9	32.5
		Actual	50.0	28.3	21.7
Late flood	Grassland	Predicted	35.1	32.7	32.2
		Actual	50.0	29.6	20.4
	Riparian woodland	Predicted	33.5	33.1	33.3
		Actual	50.0	34.0	16.0
	Secondary floodplain	Predicted	33.5	33.0	33.4
		Actual	40.0	25.7	34.3
	Tertiary floodplain	Predicted	33.6	33.4	33.0
		Actual	46.0	30.2	23.8
Rainy	Grassland	Predicted	34.0	32.5	33.5
		Actual	41.3	25.4	33.3
	Dense mopane woodland	Predicted	33.3	33.5	33.2
		Actual	46.3	22.2	31.5
	Open mopane woodland	Predicted	34.1	33.5	32.3
		Actual	45.8	20.3	33.9
	Mixed shrubland	Predicted	34.1	32.7	33.2
		Actual	45.5	23.6	30.9

## Discussion

Habitat selection and use are key topics in ecological studies [50, 51] and govern animal distribution over several scales because they can have strong influences on fitness and survival [52, 53, 54]. Most environments are naturally heterogeneous, and animals must detect and respond to variation in resource cues to identify optimal sites for particular behaviours [10, 18]. We have shown that bulk grazers, such as buffalo, use vegetation characteristics to discriminate between sites at the macro- and microhabitat levels, indicating that buffalo are responding to vegetation heterogeneity at a scale intermediate between landscape and bite levels [55]. Our study shows that buffalo engage in behavioural responses that shape movement patterns on a finer scale than previously considered, even in relatively non-selective bulk grazers, and highlights the necessity of incorporating measures of behaviour into studies of habitat use.

We did not consider herd size in our analyses, which could have contributed to site use [21], because the retrospective identification of sites from GPS data made it impossible to determine herd size during site use. Buffalo herds in the Okavango Delta ranged from 10–1500 animals, although most were in the 50–300 range, with no seasonal changes in mean herd size [49]. Individual collared animals were frequently observed in herds of varying sizes, suggesting highly dynamic fission-fusion social behaviour [55].

### Behaviour-Related Macrohabitat Use

Variation in vegetation characteristics was sufficient to identify key differences between all macrohabitats. The proportion of time that buffalo allocated to different behaviours varied between most macrohabitats, except during the rainy season, supporting our hypothesis that macrohabitat use was related to behaviour. The majority of this variation was caused by differences in the proportion of time spent resting. Cape buffalo avoided resting in secondary floodplain, presumably because this habitat was usually flooded, although forest buffalo (*Syncerus caffer nanus*) select resting sites in marshland, where they can wallow [56]. Time spent resting was greatest in woodland macrohabitats that would have provided the most shade, in keeping with findings from Ethiopia [57] and South Africa [58]. In contrast, forest buffalo in Central Africa preferentially rest in open areas, where they can maintain high levels of visual and physical contact [59], which would not be possible in dense vegetation [60]. However, vegetation characteristics other than canopy cover were better able to identify macrohabitats during the late flood season, supporting previous findings that vegetation structure was one of the least important factors in buffalo macrohabitat selection [61].

During the late flood season, forage abundance was lowest in habitats that were preferentially used for resting, as evidenced by lower biomass and height, and higher tuft dispersion. This may reflect use of sites with beneficial abiotic factors such as shade and shelter [13], which can negatively affect forage abundance [4]. Sites with low grass abundance also offer greater visibility, with an associated reduction in predation risk [62]. Resting behaviour coincides with reduced vigilance and increased vulnerability to predation [63], so resting in locations with low forage abundance may allow animals to lower their heads while maintaining their capacity to detect predators, thereby reducing perceived predation risk, an important factor in the selection of resting sites [64]. Previous studies have shown that buffalo utilise areas with intermediate levels of herbaceous biomass, possibly as a trade-off between forage intake and predation risk [65, 66, 67]. Our results suggest that visibility in resting sites may be more important than forage availability, given that the majority of a herd is either ruminating or completely inactive during resting bouts.

Behaviour-related macrohabitat use was less significant during the rainy season than the flooding seasons. This may have been caused by more uniform conditions, as indicated by the lack of significant differences between dense and open mopane woodland, with the exception of canopy cover. The only difference in proportional behaviour during the rainy season, between dense mopane woodland and mixed shrubland, was caused by buffalo spending more time grazing in the latter, where forage biomass was significantly higher. The rainy season was the most productive, when new growths of annual and perennial grasses were readily available in all macrohabitat types. This was when overall habitat selection was lowest [33], indicating that the benefits of discriminating between macrohabitats were reduced by the abundant, high quality grasses prevalent across the landscape. Similarly, buffalo in Kruger National Park, South Africa, showed lower levels of selection in areas characterised by high quality forage than in less profitable areas [55, 68]. Large-scale studies of buffalo distribution have shown that their patterns of macrohabitat use are related to vegetation greenness, a measure of quality, throughout the year [69, 70], but not to vegetation quantity [71].

### Behaviour-Related Microhabitat Use

Our results supported the hypothesis that Cape buffalo were discriminating between some microhabitats and adjusting their behaviour and associated grazing pressure to local vegetation conditions. Such a habitat use strategy facilitates exploiting heterogeneous resources that are distributed unevenly both spatially and temporally [72]. The predictive power of the microhabitat models was lower than the macrohabitat models, and differences in characteristics between microhabitats used for different behaviours were less significant than between macrohabitats. However, as at the macrohabitat scale, differences between microhabitats used for different behaviours were least apparent during the rainy season, the period when resources were most readily available.

Mean leaf table height was the characteristic that was most frequently able to distinguish between microhabitats used for different behaviours. This variable is the best indicator of visibility and, with the exception of tertiary floodplain during the late flood season, was consistently lowest in resting sites. In most cases, biomass, species richness and palatability index were highest in grazing sites. Feeding in sites with high forage abundance and quality would fulfil the intake requirements of large, herd-dwelling ruminants [25], and such a strategy has been documented in buffalo previously [73]. Herd foraging sites must support large numbers of individuals, whose interactions and movements may interfere with each other’s ability to graze selectively [74], so grazing in sites with high levels of species richness could be beneficial [57]. Large herbivores may identify optimal feeding sites based on general forage characteristics rather than selecting plants within sites because their morphological features reduce their capacity to select forage at small scales [75], although selection at the scale of individual plants has been recorded in buffalo [61].

Distance to water has a strong influence on buffalo distribution at a landscape scale, and buffalo preferentially utilise macrohabitats close to permanent water, particularly during the dry season [34, 76, 77, 78], except in the Nama-Karoo, where riverine areas are selected during the rainy season [58]. However, we did not find any consistent behavioural response to distance to water at the macro- or microhabitat scales, suggesting that it was not a factor that influenced buffalo behaviour beyond determining seasonal home ranges.

### Relative Importance of Behaviours in Macro- and Microhabitat Use

Of the 30 pair-wise differences in characteristics between microhabitats, 13, 16 and 11 involved resting, grazing and walking sites, respectively. Use of grazing sites was therefore a key process at the microhabitat scale, but there was no difference in time allocated to grazing between macrohabitats. Buffalo must consume sufficient forage to meet their requirements as bulk grazers, so the proportion of time spent foraging may not be as variable as the amount of time dedicated to resting or moving. Indeed, seasonal differences in proportional behaviour were caused by variation in walking, while the percentage of time spent grazing remained constant at approximately 45%, although buffalo in the nearby Chobe National Park only spent 37.5% of their time grazing [79].

Characteristics of resting sites were consistent across scales, whereby buffalo preferentially rested in macro- and microhabitats with high visibility. This association between resting and high visibility highlights the importance of considering behaviours other than foraging when analysing resource use patterns. While obtaining sufficient food of suitable quality is essential to survival, the high vulnerability associated with resting may explain the strong association between resting behaviour and habitat characteristics that reduce risks. The availability of appropriate resting sites may therefore have a greater impact on the spatial ecology and fitness of animals than previously considered.

We did not identify any particular macrohabitats that buffalo consistently walked through. At the microhabitat scale, walking sites were the least different from sites used for other behaviours. It is therefore likely that buffalo were selecting particular microhabitats for resting and grazing, and walking through other locations with less distinctive forage characteristics, thereby avoiding them.

### Implications for Studies of Habitat Use by Buffalo

Our findings agree with the results from previous studies on African buffalo macrohabitat use and resource requirements. Buffalo graze in sites with high levels of forage abundance and quality [57, 65, 73, 80], and rest in locations with high visibility [59, 60]. Previous studies have found that buffalo select areas with intermediate levels of biomass [66, 67], but our results suggest that they discriminate between grazing and resting sites within those areas. Buffalo rest more in woodland macrohabitats with shade, but low vegetation abundance seems to be more important than vegetation structure [61], allowing increased visibility for predator detection, but also for maintaining physical and visual contact, as in forest buffalo [59, 60]. By considering variation in vegetation characteristics within macrohabitats and their influence on behaviour, we have provided a more thorough understanding of buffalo resource requirements and the processes governing buffalo spatial ecology, and highlighted the key role of forage heterogeneity in driving behaviour [55].

### Conclusions

Large herbivores benefit from high levels of heterogeneity in macrohabitat distribution, which can buffer the effects of temporal variation in forage availability [81]. We have shown that resource heterogeneity on a smaller scale is also important in herbivore ecology, particularly during seasons when resources are restricted. Despite being bulk grazers and among the least selective herbivores [25], Cape buffalo are able to detect vegetation differences within macrohabitat types and adjust their behaviour accordingly. Herd-dwelling bulk grazers form a substantial portion of the herbivore biomass [82], and can have strong influences on vegetation. Understanding the factors that attract or repulse them from particular sites can ensure the successful management of resources. Resource use by smaller species that are capable of more precision may be affected even more strongly by disparities in the characteristics of microhabitats, so variation within, not just between, macrohabitats should be considered during studies of resource requirements.

Macrohabitats are typically defined by species composition and vegetation growth patterns, but our results have shown that measures of forage abundance may be more useful for distinguishing between functional macrohabitat types. Differences in vegetation characteristics within observer-defined macrohabitats are also important to animals [83]. We have shown that comparatively small differences between macro- and microhabitats can trigger behavioural responses. Identifying these triggers will ensure a more holistic approach to understanding animal spatial patterns. Incorporating different behaviours into scalar habitat use analyses will allow a deeper insight into animal resource requirements, which in turn can increase the efficiency of conservation and management techniques [21]. Integrating movement patterns and empirical field data increases the value of remotely-acquired GPS data [9]; in combination they play a key role in understanding animal behavioural ecology and its influence on scalar habitat use.
